# meth-SemiCancer: a cancer subtype classification framework via semi-supervised learning utilizing DNA methylation profiles

**DOI:** 10.1186/s12859-023-05272-6

**Published:** 2023-04-26

**Authors:** Joung Min Choi, Chaelin Park, Heejoon Chae

**Affiliations:** 1 Department of Computer Science, Virginia Tech, Blacksburg, USA; 2 Division of Computer Science, Sookmyung Women’s University, Seoul, Republic of Korea

**Keywords:** DNA methylation, Semi-supervised learning, Cancer subtype classification, Neural network

## Abstract

**Background:**

Identification of the cancer subtype plays a crucial role to provide an accurate diagnosis and proper treatment to improve the clinical outcomes of patients. Recent studies have shown that DNA methylation is one of the key factors for tumorigenesis and tumor growth, where the DNA methylation signatures have the potential to be utilized as cancer subtype-specific markers. However, due to the high dimensionality and the low number of DNA methylome cancer samples with the subtype information, still, to date, a cancer subtype classification method utilizing DNA methylome datasets has not been proposed.

**Results:**

In this paper, we present meth-SemiCancer, a semi-supervised cancer subtype classification framework based on DNA methylation profiles. The proposed model was first pre-trained based on the methylation datasets with the cancer subtype labels. After that, meth-SemiCancer generated the pseudo-subtypes for the cancer datasets without subtype information based on the model’s prediction. Finally, fine-tuning was performed utilizing both the labeled and unlabeled datasets.

**Conclusions:**

From the performance comparison with the standard machine learning-based classifiers, meth-SemiCancer achieved the highest average F1-score and Matthews correlation coefficient, outperforming other methods. Fine-tuning the model with the unlabeled patient samples by providing the proper pseudo-subtypes, encouraged meth-SemiCancer to generalize better than the supervised neural network-based subtype classification method. meth-SemiCancer is publicly available at https://github.com/cbi-bioinfo/meth-SemiCancer.

**Supplementary Information:**

The online version contains supplementary material available at 10.1186/s12859-023-05272-6.

## Background

Human cancer is one of the highly heterogeneous diseases driven by multiple genetic alterations and mutations [1]. Due to its divergent biological factors and relations between the genetic components, it has been a challenge to predict the prognosis and clinical outcomes of patients [2, 3]. To provide personalized treatment and precisely targeted medicine, cancers of specific tissues have been divided into subtypes based on the molecular characteristics of primary tumors. Sorlie et al. and Parker et al. [6] presented the molecular properties of breast cancer and proposed a subtyping system named PAM50 classifying breast cancer into five intrinsic subtypes [4, 5]. Abeshouse et al. revealed novel molecular features related to primary prostate cancer and established a molecular taxonomy of it. The premise is that cancer patients within the same subtype would share similar responses to therapy and prognostic outcome, where subtype identification could lead to a better diagnosis of patients and help to reveal and understand the tumor biology system [7].

Based on the defined cancer subtyping system, a cancer diagnosis has been improved. In the early stages, traditional diagnosis heavily relies on manual inspections from human clinical expertise causing expensive costs and relatively low accuracy [8, 9]. To address these issues, computational methods utilizing gene expression profiles have been presented, where machine learning-based approaches including neural networks have been widely applied for cancer subtype classification [10–12]. Recently, studies have shown that epigenetic alterations play key roles in cancer development, where epigenetic changes are involved in the earliest phases of tumorigenesis and tumor promotion [13, 14]. DNA methylation is the most extensively studied epigenetic mechanism. Several studies suggested that hypermethylation of CpG islands regions leads to transcriptional silencing, which is a key factor in tumor growth [15, 16]. Epigenetic analyses have identified aberrant DNA methylation signature patterns are related to the molecular subtypes of cancers [14], suggesting a potential to be utilized for subtype-specific markers [17]. For example, Holm et al. [18] revealed that the molecular subtypes of breast cancer display specific methylation profiles, suggesting that methylation may play a key role in the development of breast cancers. Bediaga et al. [14] also provided evidence that distinct DNA methylation profiles enable the prediction of breast cancer subtype, prognostication, and the therapeutic stratification of the patients. Zhang et al. [19] identified methylation patterns of 8 CpGs related to the molecular subtypes of the prostate cancer, and Ylitalo et al. [20] also investigated a promoter methylation signature which predicts the activity of tumor and the patient prognosis. Chen et al. [21] validated that DNA methylation-based classification can be used for identifying the distinct subtypes of renal cell carcinoma.

DNA methylome holds much promise as biomarker in cancer, however, several challenges remain in developing a method to improve the diagnosis of cancer utilizing the methylation dataset. Due to the much higher dimensionality and complexity with a large number of CpGs compared to other biological datasets, the traditional machine learning models suffer difficulty in the training phase. Moreover, to train those models for subtype prediction of each cancer patient, a large amount of dataset having subtype label information is needed to prevent the overfitting issue. However, the number of public cancer methylome datasets is limited to be utilized for research, and for most of the datasets, the subtype information for each patient is not provided, which makes it difficult for the current classification models to utilize those for training.

In recent, to alleviate the need for labeled data, semi-supervised learning (SSL) has been presented, which allows a model to leverage unlabeled data [22]. Since unlabeled data can be obtained with the low cost of human labor, a plethora of SSL methods have been presented for deep neural networks [23–26]. One of the popular SSL approaches is to produce pseudo labels for unlabeled samples based on the model’s prediction and feed them as input to train against [26]. SSL utilizes those unseen data to better generalize the model and avoid the overfitting of the training dataset, which has shown improvement in image classification performance. SSL has also been applied to gene expression datasets. scSemiCluster presented a framework to improve cell type classification combining SSL with domain adaptation for single-cell RNA-sequencing (scRNA-seq) datasets [27], and SemiRNet implemented cell identification tool utilizing the unlabeled scRNA-seq cells based on the semi-supervised recurrent convolutional neural network model [28]. These studies have shown the application of SSL has the potential to address the lack of labeled data issue in the biological domain.

In this paper, we proposed a cancer subtype classification framework based on semi-supervised learning, meth-SemiCancer, which utilizes the unlabeled DNA methylome cancer dataset for training. Our model was first pre-trained with the DNA methylation dataset having the subtype information, and a pseudo-label was assigned to the unlabeled sample. Utilizing those, meth-SemiCancer was re-trained by optimizing the weighted cross-entropy loss using both labeled and pseudo-labeled datasets, where the pseudo-label was updated in each iteration. The performance of meth-SemiCancer was compared to that of several supervised machine learning (ML) classifiers. meth-SemiCancer improved the cancer subtype prediction performance, by providing proper pseudo-labels on the unlabeled cancer methylome datasets via SSL.

## Methods

**Fig. 1:**
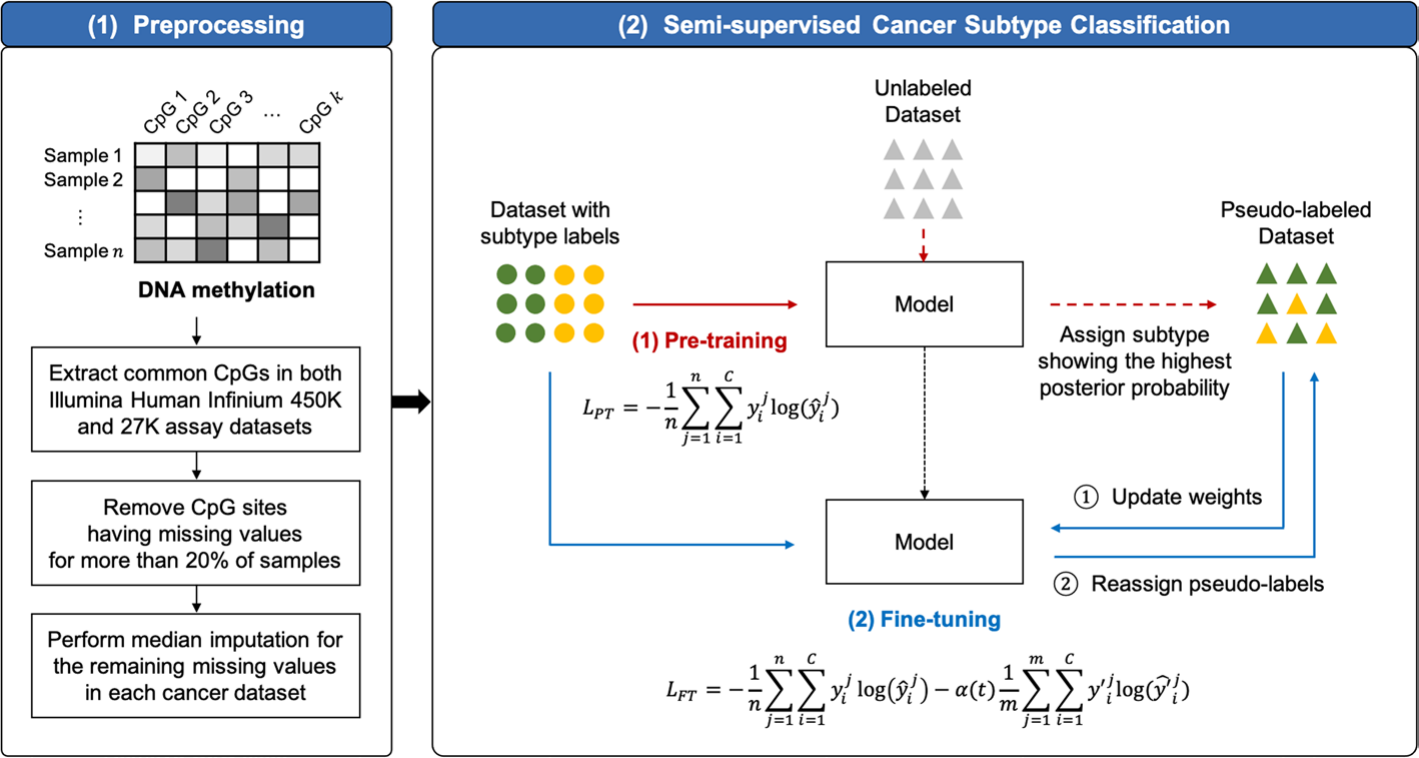
Illustration of the proposed cancer subtype classification framework via Semi-supervised learning utilizing DNA methylation profiles

Our meth-SemiCancer framework consists of two phases: (1) preprocessing and (2) semi-supervised cancer subtype classification. The workflow of our model is shown in Fig. 1.

### Data collection

To pretrain meth-SemiCancer, DNA methylome cancer datasets were collected from TCGA [29], where primary solid tumor tissue samples measured by Illumina Human Infinium 450K and 27K assay were downloaded. Six cancers with subtypes defined, Breast invasive carcinoma (BRCA), Colon Adenocarcinoma (COAD), Glioblastoma Multiforme (GBM), Prostate adenocarcinoma (PRAD), Renal cell carcinoma (RCC), and Thyroid carcinoma (THCA) were used. Subtype information for each cancer was retrieved from [5, 30–34] respectively. We also obtained DNA methylation datasets for the above six cancers from Gene Expression Omnibus (GEO) [35], where these datasets did not provide subtype information, utilizing them as unlabeled datasets. The number of samples and subtype information for each cancer are shown in Tables 1 and 2.

**Table 1:** Datasets used for training meth-SemiCancer

Cancer	Labeled datasets (with the subtype information)	Total	Unlabeled datasets (without the subtype information)			Total
BRCA	TCGA-BRCA	1056	GSE72251 GSE20712 GSE72245	GSE75067 GSE69914 GSE156968	GSE66695 GSE58999 GSE141441	1225
COAD	TCGA-COAD	475	GSE118970 GSE27130	GSE131013 GSE57342	GSE68060 GSE77954	436
GBM	TCGA-GBM	375	GSE200647 GSE195684	GSE195640 GSE188547	GSE121722	643
PRAD	TCGA-PRAD	333	GSE115413 GSE83917	GSE112047 GSE26126	GSE76938	400
RCC	TCGA-KICH, KIRC, KIRP	900	GSE61441 GSE105260	GSE126441	GSE113501	258
THCA	TCGA-THCA	496	GSE97466	GSE86961	GSE72729	156

**Table 2:** List of cancers with subtypes used in this study

Cancer	Subtypes
BRCA	LumA, LumB, Her2, Basal, Normal-like
COAD	CMS1, CMS2, CMS3, CMS4
GBM	Mesenchymal, Classic, LGm6, G-CIMP high, G-CIMP low
PRAD	ERG, ETV1, ETV4, SPOP, other
RCC	KICH, KIRC, KIRP
THCA	Follicular, Classical 1, Classical 2, CpG island methylated

### Preprocessing

As Illumina Human Infinium 27K assay technique only allows to measure the methylation status of 27K CpG sites due to the limitation of the technique, the datasets processed based on 27K assay results in having the subset of the features in 450K dataset. To utilize both DNA methylome datasets measured by Illumina Human Infinium 450K and 27K assay for better training and generalized optimization of the classification model, common features were used for further steps. To reduce the bias caused by a high frequency of missing values during model training, CpG sites having more than 20% missing values were removed, and the imputation was performed for the remaining missing values.

For the selection of the imputation strategy, we investigated the impact of the imputation method on the cancer subtype prediction, we performed an experiment based on the breast cancer datasets. For the datasets collected from TCGA and GEO repository, the most widely used imputation strategies, mean, median and KNN imputation were applied. By comparing the subtype classification performance based on Tenfold cross validation, the meth-SemiCancer showed the best performance when the median imputation was applied, leading to the conclusion to adopt the median imputation (Additional file 1: S1). Preprocessing was performed for each dataset, and the number of 11,827 (BRCA), 23,378 (COAD), 17,660 (GBM), 10,329 (PRAD), 13,493 (RCC), and 22,198 (THCA) CpGs were remained after preprocessing.

### Semi-supervised cancer subtype classification

The meth-SemiCancer model is constructed based on a neural network with semi-supervised learning, where the proposed model is trained using both labeled and unlabeled methylome datasets in a supervised fashion for cancer subtype prediction. The overall architecture of our model is shown in Additional file 1: S2. Given a set of original input data $x \in {{\textbf {R}}}^k$, where *k* is the dimension of the input data, the meth-SemiCancer consists of two fully connected layers, each with 1000 and 500 hidden nodes, followed by a softmax layer, which estimates the posterior probability of $i_{th}$ cancer subtype through the softmax function $S_i$:

1 $$\begin{aligned} h&= f(W_1x+b_1), \quad a = f(W_2h+b_2) \end{aligned}$$

2 $$\begin{aligned} S_i&= {{\exp (a_i)} \over {\sum _{j=1}^C \exp (a_j)}} \end{aligned}$$

where $W_1$, $W_2$ are the weights, $b_1$, $b_2$ are the bias, *f* is a ELU [36] activation function, and *C* is the number of subtypes. First, meth-SemiCancer is pre-trained using the labeled datasets to initialize the weights and learn the hidden representations. After pre-training, pseudo-labels for the unlabeled datasets were obtained by assigning the cancer subtype showing the highest posterior probability, which were used as if they were true labels. Fine-tuning was performed utilizing both the labeled and unlabeled datasets, where the pseudo-labels were newly calculated for every weight update.

For optimization, both pre-training (PT) and fine-tuning (FT) phases use the cross-entropy as a loss function to minimize, but slightly in a different manner. During the pre-training phase, meth-SemiCancer is trained based on the standard cross-entropy loss ($\mathcal {L}_{PT}$) utilizing only a labeled dataset. For fine-tuning, the loss function needs to be balanced between the labeled and unlabeled datasets, since the number of each dataset is different and there could be batch effects and variations caused by the unlabeled DNA methylation cancer datasets collected from different experimental settings and sequencing protocols. Following the suggestion from [26] to use a weighted cross-entropy loss for the labeled and unlabeled datasets, a coefficient $\alpha (t)$ was introduced in the loss function of fine-tuning for training balance as follows:

3 $$\begin{aligned} \mathcal {L}_{PT}&= -\frac{1}{n}\sum _{j=1}^{n}\sum _{i=1}^{C}y_i^j\log (\hat{y}_i^j) \end{aligned}$$

4 $$\begin{aligned} \mathcal {L}_{FT}&= -\frac{1}{n}\sum _{j=1}^{n}\sum _{i=1}^{C}y_i^j\log (\hat{y}_i^j) -\alpha (t)\frac{1}{m}\sum _{j=1}^{m}\sum _{i=1}^{C} {y'}_i^j\log (\hat{y'}_i^j) \end{aligned}$$

where *n* is the number of samples in the labeled dataset, *m* for the unlabeled dataset, *y* ($\hat{y}$) is the true (model predicted, respectively) subtype probability distribution for the labeled dataset, and $y'$ ($\hat{y}'$) is the pseudo-subtype (model predicted) probability distribution for the unlabeled dataset. For a coefficient $\alpha (t)$ to balance the training loss between the labeled and unlabeled datasets, it was slowly increased to help the optimization process to avoid poor local minima [37], since a high value of $\alpha (t)$ could interfere training by labeled dataset [26]:

5 $$\begin{aligned} \alpha (t)=\left\{ \begin{array}{ll} 0, &{} t<T_{1}\\ \frac{t-T_{1}}{T_{2}-T_{1}}\alpha _{f}, &{} T_{1}\le t<T_{2}\\ \alpha _{f}, &{} T_{2}\le t\\ \end{array}\right. \end{aligned}$$

where t is current epoch with $T_{1}=100, T_{2}=200$. During the training phases, the adaptive moment estimation (Adam) optimization algorithm [38] was used, the dropout rate was set to 0.7, and L2 regularization was applied to prevent overfitting. The hyperparameters in meth-SemiCancer including the learning rate, training epochs, and the alpha value were optimized based on the TCGA-BRCA dataset, where the training and testing datasets were split randomly with the ratio of 9:1. For each parameter, the experiment was repeated five times, and the combination of the hyperparameters showing the highest average accuracy was selected. The learning rate and training epoch was set to 1e−5 and 1500 for pre-training, and 1e−3 and 3000 for fine-tuning. The $\alpha _f$ was set to 0.05. Accuracy results from the experiments with different parameter settings are shown in the Additional file 1: S3. Our proposed model was built by Tensorflow library (Version 1.8.0).

## Results

### Performance evaluation of meth-SemiCancer

To evaluate the performance of meth-SemiCancer for cancer subtype classification, we compared the proposed model with the baseline methods. To the best of our knowledge, this is the first study presenting the neural network-based cancer subtype classification framework utilizing the DNA methylation datasets, the widely-used ML-based classifiers were compared with our meth-SemiCancer: Support vector machine (SVM) [39], Random Forest (RF) [40], K-nearest neighbors (KNN) [41], Naive Bayes (NB) [42], and Decision Tree (DT) [43]. SVM is a supervised learning algorithm that identifies a hyperplane to create a decision boundary classifying the data points to each class by maximizing the margin between the classes. DT constructs a tree-structured classification model based on the set of discrete rules from the training dataset. Starting at the root node, it follows the appropriate branches by comparing with the decision rule and based on the terminal node the data point will be assigned to one of the classes. RF is the extension of DT, which is an ensemble learning method aggregating the output from multiple DTs to derive a final result. The KNN is a non-parametric classifier predicting a class of each data point based on the neighbors within the close distance, and the NB classifies the data based on Bayes’ theorem with the assumption of conditional independence between the features.

Following the same optimization procedure used for meth-SemiCancer, the baseline methods were optimized based on the TCGA-BRCA dataset, where the training and testing datasets were randomly split to the ratio of 9:1. Grid search was adopted for the model tuning. For each combination of hyperparameters, the experiment was repeated five times, and the parameters showing the highest average accuracy for the testing dataset were selected (Additional file 1: S3). The optimized hyperparameter settings for each classifier are as follows: SVM (kernel = rbf, C = $2^5$, gamma = $2^{-11}$), RF (criterion = gini, estimators = 100, min_samples_leaf = 1), KNN (weights = distance, n_neighbors = 10), DT (criterion = entropy, min_samples_leaf = 3). For each cancer subtype, Tenfold cross validation was performed using TCGA datasets and the unlabeled datasets obtained from GEO repository were used for meth-SemiCancer during the fine-tuning phase. For the evaluation metrics, the accuracy, precision, recall, F1-score, Matthews correlation coefficient (MCC), and Cohen’s Kappa were adopted. The accuracy is a ratio of the closeness between the prediction and the true labels. The recall and the precision refer to the proportion of actual positive classes that are correctly predicted positive and the proportion of predicted positive classes that are correctly assigned as real positive, respectively [44], here, the ’macro’ option was used. The F1-score is the harmonic average of the precision and recall between 0 and 1, where we used the ’weighted’ option for an average parameter to deal with the multi-class tasks. The MCC considers the true and false positives and negatives to overcome the class imbalance [45], and Kappa measures the reliability between the predicted class and true class.

For GBM and THCA cancer datasets from TCGA, some subtypes had less than 40 samples each, which could lead to the underfitting during the training phases in the classifiers (LGm6, G-CIMP low/high for GBM and Classical 1, CpG island methylated for THCA). To address this issue, we generated a simulated DNA methylation dataset with a size of 100 samples only for those subtypes using methCancer-gen [46] tool, which is a DNA methylome dataset generator for user-specified cancer type based on conditional variational autoencoder. The generated samples were only added to the training phases in our meth-SemiCancer and other comparison methods.

**Fig. 2:**
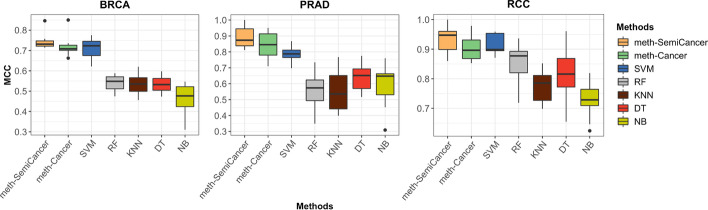
Performance comparison of meth-SemiCancer with the baseline methods based on Tenfold cross validation (CV). The Boxplot for each cancer shows the distribution of MCCs for the classifiers in Tenfold CV of each cancer subtype prediction

For each cancer, the boxplots were plotted to compare the distribution of MCCs for each classier in Tenfold cross validation (Fig. 2, Additional file 1: S4). meth-SemiCancer outperformed the other ML-based classifiers with the highest average MCC of 0.755 (BRCA), 0.689 (COAD), 0.987 (GBM), 0.888 (PRAD), 0.939 (RCC), and 0.986 (THCA) for each cancer, respectively. The SVM showed the second-best performance showing an average MCC of 0.712 (BRCA), 0.561 (COAD), 0.964 (GBM), 0.783 (PRAD), 0.917 (RCC), and 0.940 (THCA). From the comparison of the boxplots, our proposed method achieved the best median MCC for all cancer subtypes, presenting the superiority in cancer subtype classification tasks. In case of BRCA, COAD, GBM, and THCA, meth-SemiCancer plotted shortest boxes compared to the other methods, exhibiting the less variability in subtype classification performance which can be also shown as the stability of our method. SVM also had the stable performance for most of the cancer types, however, in case of COAD, it showed the severe performance change, obtaining the lowest MCC of 0.407 and 0.681 for the highest MCC. Our model also achieved the highest average accuracy and F1-score for all six cancers, compared to the other methods (Additional file 1: S5). Moreover, we investigated whether SSL utilizing the unlabeled dataset with pseudo-labels helps to improve the cancer subtype prediction performance. We implemented the variant of meth-SemiCancer removing the fine-tuning phase, denoted as ’meth-Cancer’, and compared the performance. The result showed that when the model was trained based on the SSL approach, the average classification performance increased for all three cancers across all the evaluation metrics, which achieved the 5.8% of performance improvement in MCC for PRAD and 2.4% in F1-score for BRCA cancer subtype prediction. This could be also seen from the comparison of boxplots in Fig. 2, where the median MCC improved significantly in RCC and slight improvement in BRCA and PRAD.

### Validation of the cancer subtype prediction for unlabeled dataset

We tested whether the pseudo-labeling approach in SSL accurately identifies the cancer subtype of the unlabeled dataset. In this experiment, we divided the labeled cancer datasets obtained from TCGA into the ratio of 8:1:1 and utilized each part as a pre-training (labeled), fine-tuning (unlabeled), and testing dataset, respectively. For the dataset used during the fine-tuning phase, we assumed them as unlabeled for model training, however, since we already had the actual subtype information of those, we could validate whether the meth-SemiCancer can assign a proper pseudo-subtype label for each cancer patient. Fivefold cross validation was performed and the average accuracy was measured for the unlabeled and testing datasets. From the result (Table 3), meth-SemiCancer could predict the subtype for most of the cancer patients in unlabeled datasets during fine-tuning with the average accuracy of 0.823 (BRCA), 0.921 (GBM), 0.916 (PRAD), 0.964 (RCC), and 0.904 (THCA). We could also see that the classification accuracy for the testing dataset improved after fine-tuning was performed, compared to the accuracy when only pre-training was done. In the case of COAD cancer, the average accuracy of the unlabeled dataset was a bit low showing an accuracy of 0.679, but still utilizing those datasets for better optimization of the model, the prediction performance for the testing dataset after fine-tuning improved.

**Table 3:** Average classification performance results performing Fivefold cross validation based on the TCGA datasets

Cancer	Unlabeled dataset		Testing dataset (pre-training)		Testing dataset (fine-tuning)	
	Accuracy	F1-score	Accuracy	F1-score	Accuracy	F1-score
BRCA	0.823	0.801	0.800	0.805	0.827	0.817
COAD	0.679	0.678	0.749	0.747	0.757	0.756
GBM	0.921	0.922	0.881	0.884	0.908	0.904
PRAD	0.916	0.901	0.827	0.877	0.891	0.884
RCC	0.964	0.960	0.947	0.949	0.956	0.958
THCA	0.904	0.911	0.842	0.854	0.899	0.894

### Investigating the effect of the amount of unlabeled dataset for fine-tuning

The success of SSL approaches in neural networks is attributable to allowing a model to leverage the unlabeled dataset and encouraging a model to generalize better to unseen data [23]. It has brought a significant performance improvement with low cost in several domains including computer vision applications, where the larger amount of unlabeled dataset could be utilized, the more generalized performance is provided from the model training. However, compared to the image datasets, the biological datasets, in particular, the cancer patient samples are having much difficulty in generalization due to their tumor heterogeneity. In this experiment, we investigated whether the larger amount of unlabeled cancer dataset used for fine-tuning could lead to the subtype prediction performance. First, we generated a simulated DNA methylation dataset for each cancer using methCancer-gen [46] tool by increasing a sample size from 400 to 1000 samples, which is a DNA methylome dataset generator for user-specified cancer type based on conditional variational autoencoder. Tenfold cross validation was performed using the datasets from TCGA, where the training dataset was used for pre-training the meth-SemiCancer, and methCancer-gen training, and the simulation samples were used for the fine-tuning phase of meth-SemiCancer. Here, we performed the experiment using BRCA, PRAD, and RCC cancer datasets. The results (Table 4, Additional file 1: S6) have shown that the average classification performance slightly increased when the model was fine-tuned with the larger amount of unlabeled datasets. For example, BRCA showed the average f1-score increase from 0.805 to 0.825, and PRAD also achieved an improvement from 0.877 to 0.899, when the model was trained utilizing 1000 simulated samples.

In addition, we tested the same experiment using the real studies dataset. Tenfold cross validation was performed using TCGA datasets, where the subset of the datasets from GEO repository was used for fine-tuning (Table 1). The testing performance of meth-SemiCancer was measured with the increase of the subset sample sizes by randomly selecting 40%, 60%, 80% and using all the datasets. The experiment obtained a similar result from the above, where the model showed the highest classification performance, when utilizing all the GEO datasets for fine-tuning, compared to using the subset of the datasets (Table 4, Additional file 1: S6).

**Table 4:** Average F1-score results under different sample sizes for pseudo-labeling during fine-tuning based on Tenfold cross-validation

Sample size	Simulation dataset					Real-studies dataset				
	0	400	600	800	1000	0%	40%	60%	80%	100%
BRCA	0.814	0.820	0.822	0.821	0.825	0.814	0.824	0.822	0.818	0.833
PRAD	0.872	0.882	0.890	0.885	0.899	0.872	0.913	0.901	0.907	0.915
RCC	0.948	0.981	0.979	0.977	0.976	0.948	0.967	0.969	0.974	0.967

### The effect of the confidence threshold for pseudo-labeling

Recent studies have introduced a confidence threshold, which is a trade-off between the quality and the quantity of pseudo-labels [25, 47]. By only utilizing the pseudo-labeled samples which obtained the higher prediction probability for the cancer subtype, we could prevent the model from being impeded by noisy pseudo-labeled examples. The high threshold values allow high-quality unlabeled samples to contribute to the unlabeled loss, but it has a chance to reduce the quantity of pseudo-labels. We performed an experiment using BRCA datasets to see the impact of this confidence threshold when introduced in our meth-SemiCancer. For every iteration during the fine-tuning phase, the pseudo-labeled samples higher than the confidence threshold were selected and only those were used for training the model. Tenfold cross validation was performed based on the TCGA datasets, and the GEO datasets were used for fine-tuning phase.

When introducing the higher confidence threshold, a larger number of unlabeled samples were filtered out during the early fine-tuning phase (Additional file 1: S7). But, as the training epoch increases, our model learns to produce high-confidence predictions, utilizing all the unlabeled samples for optimization at the end of the training. This led to similar average performance results of meth-SemiCancer between the different confidence thresholds (Table 5). Our model also did not show a significant difference compared to the previous result not using the threshold, consistent with the study [25], where the authors proposed to introduce the confidence threshold in the SSL approach, however, the approach did not show performance improvement. In addition, we tested our model with the threshold to other cancers, however, depending on the cancer type, the threshold criteria vary and the amount of dataset that can be utilized decreased significantly. From these issues, meth-SemiCancer did not include the confidence threshold mechanism for the experiments, but still, we provided an option to enable the user-specified confidence threshold in our meth-SemiCancer.

**Table 5:** Average BRCA subtype classification performance results under different confidence thresholds for pseudo-labeling based on Tenfold cross-validation

Confidence threshold	0.5	0.6	0.7	0.8	0.9
Accuracy	0.836	0.839	0.842	0.844	0.842
F1-score	0.821	0.826	0.828	0.834	0.831
MCC	0.747	0.743	0.753	0.751	0.745
Recall	0.729	0.719	0.719	0.729	0.694
Precision	0.793	0.782	0.794	0.798	0.768
Kappa	0.742	0.737	0.746	0.742	0.735

## Discussion and conclusions

Although the high-throughput sequencing technologies to measure the DNA methylation profiles have been advanced, still, the public cancer datasets with the cancer subtype information are still limited to be utilized for the research due to the cost-efficiency. It has been difficult to develop a cancer subtype classification model utilizing the DNA methylome datasets which have a potential for biomarkers of subtype identification due to the lack of cancer samples with the subtype labels. Recently, several studies have presented a semi-supervised learning approach to leverage the unlabeled dataset and have proved performance improvement with the low cost of data collection in computer vision applications. We proposed meth-SemiCancer, a cancer subtype prediction framework based on semi-supervised learning utilizing DNA methylation profiles.

We first evaluated the performance of meth-SemiCancer with the standard ML-based classification methods. The performance comparison with the baseline classifiers shows that meth-SemiCancer provides more accurate subtype prediction results across all three cancers. meth-SemiCancer was also compared with the supervised version of the model removing the fine-tuning phase, and it is proved that retraining the model with the unlabeled datasets assigning the pseudo-subtype labels has the potential to generalize the model and prevent the overfitting to improve the subtype classification.

Moreover, we validated whether the meth-SemiCancer generates the proper pseudo-subtype label for the unlabeled dataset during the fine-tuning phase. From our experiment, meth-SemiCancer could learn to identify the cancer subtypes during pretraining and accurately predict most of the cancer patients without the subtype information to utilize those for retraining.

In addition, we investigated the effect of the amount of unlabeled dataset and the confidence threshold for fine-tuning phase. First, we increased the sample size of unlabeled datasets used for pseudo-labeling and measured the performance changes. The performance of meth-SemiCancer slightly improved by increasing the number of unlabeled samples for both simulation and real-studies datasets, indicating that the larger number of unlabeled datasets used for pseudo-labeling, the higher performance improvement could be achieved. However, since we could not obtain a large number of real methylation cancer samples for pseudo-labeling, a significant performance improvement could not be shown from the result. In the case of the simulation datasets, those datasets were also generated based on the same training dataset used for pre-training, there has been a limitation for a model to be better generalized for new datasets. The experiment for testing the impact of the confidence threshold was also performed, but it did not lead to a significant difference in the performance compared to that of not using the threshold, where we provided it as an option for the user.

Overall, these findings indicate that meth-SemiCancer can enable the DNA methylation cancer datasets without subtype information to be utilized for improving the cancer subtype classification by providing the proper annotation of the subtypes. We also expect that our proposed model will facilitate the in-depth biological findings by supporting the study of methylation signatures to differentiate the subtypes of complex cancer.

One limitation of meth-SemiCancer is that it makes an implicit assumption that the unlabeled datasets used for the pseudo-labeling approach in SSL have been prepared with the correction of the batch effect. In future work, we plan to extend our meth-SemiCancer framework to automatically detect and minimize the batch effects in DNA methylation data during the model training phases.

## Supplementary Information

**Additional file1. S1:** Average performance results of meth-SemiCancer for breast cancer subtype classification based on the different imputation strategies, conducting 10-fold cross validation. **S2:** The overall architecture of meth-SemiCancer. **S3:** Optimization results of meth-SemiCancer and the baseline methods with the different combination of the parameters. **S4:** Performance comparison of meth-SemiCancer with the baseline methods based on 10-fold cross validation for COAD, GBM, and THCA cancer. **S5:** The average accuracy, weighted F1-score, and Matthews correlation coefficient (MCC), precision, recall, and Cohen's Kappa results of meth-SemiCancer and the baseline methods from the performance evaluation based on 10-fold cross validation. **S6:** The average accuracy results of meth-SemiCancer under different sample sizes for pseudo-labeling during fine-tuning based on 10-fold cross-validation. **S7:** The number of unlabeled samples utilized during training the meth-SemiCancer for each fine-tuning epoch based on the different confidence threshold.

## Data Availability

TCGA methylation datasets are available from GDC Data Portal (https://portal.gdc.cancer.gov/) and other methylation samples used in the experiment are available from GEO repository (https://www.ncbi.nlm.nih.gov/geo/) with the GEO accession of GSE72251, GSE20712, GSE72245, GSE75067, GSE69914, GSE156968, GSE66695, GSE58999, GSE141441, GSE115413, GSE83917, GSE112047, GSE26126, GSE76938, GSE61441, GSE105260, GSE126441, GSE113501, GSE118970, GSE27130, GSE131013, GSE57342, GSE68060, GSE77954, GSE200647, GSE195684, GSE195640, GSE188547, GSE121722, GSE97466, GSE86961, and GSE72729. meth-SemiCancer is publicly available at https://github.com/cbi-bioinfo/meth-SemiCancer.

## Abbreviations

SSL: Semi-supervised learning
scRNA-seq: Single cell RNA-sequencing
ML: Machine learning
BRCA: Breast invasive carcinoma
COAD: Colon adenocarcinoma
GBM: Glioblastoma multiforme
PRAD: Prostate adenocarcinoma
RCC: Renal cell carcinoma
THCA: Thyroid carcinoma
GEO: Gene expression omnibus
PT: Pre-training
FT: Fine-tuning
Adam: Adaptive moment estimation
SVM: Support vector machine with rbf kernel
RF: Random forest
KNN: K-nearest neighbors
NB: Naive Bayes
DT: Decision tree
MCC: Matthews correlation coefficient
