# Supplementary material for: meth-SemiCancer: a cancer subtype classification framework via semi-supervised learning utilizing DNA methylation profiles
Source: BMC Bioinformatics. 2023 Apr 26;24:168. doi: 10.1186/s12859-023-05272-6 (PMC10131478; doi:10.1186/s12859-023-05272-6)
Supplement: Supplementary file 1 — Additional file1. S1: Average performance results of meth-SemiCancer for breast cancer subtype classification based on the different imputation strategies, conducting 10-fold cross validation. S2: The overall architecture of meth-SemiCancer. S3: Optimization results of meth-SemiCancer and the baseline methods with the different combination of the parameters. S4: Performance comparison of meth-SemiCancer with the baseline methods based on 10-fold cross validation for COAD, GBM, and THCA cancer. S5: The average accuracy, weighted F1-score, and Matthews correlation coefficient (MCC), precision, recall, and Cohen's Kappa results of meth-SemiCancer and the baseline methods from the performance evaluation based on 10-fold cross validation. S6: The average accuracy results of meth-SemiCancer under different sample sizes for pseudo-labeling during fine-tuning based on 10-fold cross-validation. S7: The number of unlabeled samples utilized during training the meth-SemiCancer for each fine-tuning epoch based on the different confidence threshold. [file 12859_2023_5272_MOESM1_ESM.pdf]

Supplementary Material S1.

Average performance results of meth-SemiCancer for breast cancer subtype classification based on the different imputation strategies, conducting 10-fold cross validation.

	Median imp	Mean imp	KNN imp
Accuracy	**0.841**	0.838	0.833
F1-score	**0.829**	0.828	0.824
MCC	**0.752**	0.750	0.738
Recall	0.725	**0.732**	0.722
Precision	**0.822**	0.803	0.803
Kappa	**0.743**	**0.743**	0.734

Supplementary Material S2. The overall architecture of meth-SemiCancer.

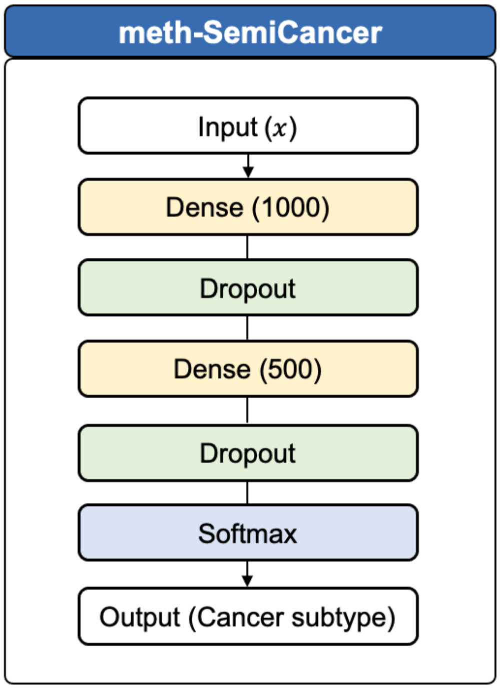

Loss function for each training phase

(1) Pre-training

$$L_{PT} = -\frac{1}{n}\sum_{j=1}^{n}\sum_{i=1}^{C} y_i^j \log(\hat{y}_i^j)$$

(2) Fine-tuning

$$L_{FT} = -\frac{1}{n}\sum_{j=1}^{n}\sum_{i=1}^{C} y_i^j \log(\hat{y}_i^j) - \alpha(t)\frac{1}{m}\sum_{j=1}^{m}\sum_{i=1}^{C} {y'}_i^j \log(\widehat{y'}_i^j)$$

Supplementary Material S3.

Optimization results of meth-SemiCancer and the baseline methods with the different combination of the parameters. Grid search was adopted for the model tuning, and the hyperparameters showing the best accuracy were selected.

meth-SemiCancer

# of hidden nodes	Pre-training		Fine-tuning		Dropout	Alpha	Accuracy
	Learning rate	Trining epoch	Learning rate	Trining epoch			
1000-500	1e-5	1000	1e-3	2000	0.7	0.001	0.8018
1000-500	1e-5	1000	1e-3	2000	0.7	0.005	0.8113
1000-500	1e-5	1000	1e-3	2000	0.7	0.01	0.8113
1000-500	1e-5	1000	1e-3	2000	0.7	0.05	0.8018
1000-500	1e-5	1000	1e-3	2000	0.7	0.1	0.8018
1000-500	1e-5	1000	1e-3	2000	0.7	0.5	0.8018
1000-500	1e-3	1000	1e-3	2000	0.7	0.001	0.8113
1000-500	1e-3	1000	1e-3	2000	0.7	0.005	0.8113
1000-500	1e-3	1000	1e-3	2000	0.7	0.01	0.8207
1000-500	1e-3	1000	1e-3	2000	0.7	0.05	0.8207
1000-500	1e-3	1000	1e-3	2000	0.7	0.1	0.8113
1000-500	1e-3	1000	1e-3	2000	0.7	0.5	0.8113
1000-500	1e-5	1500	1e-3	3000	0.7	0.001	0.8301
1000-500	1e-5	1500	1e-3	3000	0.7	0.005	0.8301
1000-500	1e-5	1500	1e-3	3000	0.7	0.01	0.8301
**1000-500**	**1e-5**	**1500**	**1e-3**	**3000**	0.7	**0.05**	**0.8490**
1000-500	1e-5	1500	1e-3	3000	0.7	0.1	0.8301
1000-500	1e-5	1500	1e-3	3000	0.7	0.5	0.8207
1000-500	1e-3	1500	1e-3	3000	0.7	0.001	0.8207
1000-500	1e-3	1500	1e-3	3000	0.7	0.005	0.8207
1000-500	1e-3	1500	1e-3	3000	0.7	0.01	0.8301
1000-500	1e-3	1500	1e-3	3000	0.7	0.05	0.8301
1000-500	1e-3	1500	1e-3	3000	0.7	0.1	0.8301
1000-500	1e-3	1500	1e-3	3000	0.7	0.5	0.8207

Support vector machine (SVM)

Kernel	Penalty parameter (C)	RBF kernel coeff (Gamma)	Accruacy
RBF	2^{-5}	2^{-15}	0.5377
RBF	2^{-5}	2^{-13}	0.5377
RBF	2^{-5}	2^{-11}	0.5377
RBF	2^{-5}	2^{-9}	0.5377
RBF	2^{-5}	2^{-7}	0.5377
RBF	2^{-5}	2^{-5}	0.5377
RBF	2^{-5}	2^{-3}	0.5377
RBF	2^{-5}	2^{-1}	0.5377
RBF	2^{-5}	2^{1}	0.5377
RBF	2^{-5}	2^{3}	0.5377
RBF	2^{-3}	2^{-15}	0.5377
RBF	2^{-3}	2^{-13}	0.5377
RBF	2^{-3}	2^{-11}	0.5755
RBF	2^{-3}	2^{-9}	0.6509
RBF	2^{-3}	2^{-7}	0.6321
RBF	2^{-3}	2^{-5}	0.5377
RBF	2^{-3}	2^{-3}	0.5377
RBF	2^{-3}	2^{-1}	0.5377
RBF	2^{-3}	2^{1}	0.5377
RBF	2^{-3}	2^{3}	0.5377
RBF	2^{-1}	2^{-15}	0.5377
RBF	2^{-1}	2^{-13}	0.5849
RBF	2^{-1}	2^{-11}	0.6509
RBF	2^{-1}	2^{-9}	0.7264
RBF	2^{-1}	2^{-7}	0.6604
RBF	2^{-1}	2^{-5}	0.5566
RBF	2^{-1}	2^{-3}	0.5377
RBF	2^{-1}	2^{-1}	0.5377
RBF	2^{-1}	2^{1}	0.5377
RBF	2^{-1}	2^{3}	0.5377
RBF	2^{1}	2^{-15}	0.5849
RBF	2^{1}	2^{-13}	0.6509
RBF	2^{1}	2^{-11}	0.7547
RBF	2^{1}	2^{-9}	0.8019
RBF	2^{1}	2^{-7}	0.8208
RBF	2^{1}	2^{-5}	0.5849
RBF	2^{1}	2^{-3}	0.5377

RBF	2^{1}	2^{-1}	0.5377
RBF	2^{1}	2^{1}	0.5377
RBF	2^{1}	2^{3}	0.5377
RBF	2^{3}	2^{-15}	0.6509
RBF	2^{3}	2^{-13}	0.7547
RBF	2^{3}	2^{-11}	0.8019
RBF	2^{3}	2^{-9}	0.8302
RBF	2^{3}	2^{-7}	0.8019
RBF	2^{3}	2^{-5}	0.5849
RBF	2^{3}	2^{-3}	0.5377
RBF	2^{3}	2^{-1}	0.5377
RBF	2^{3}	2^{1}	0.5377
RBF	2^{3}	2^{3}	0.5377
RBF	2^{5}	2^{-15}	0.7642
RBF	2^{5}	2^{-13}	0.8113
**RBF**	$\mathbf{2^{5}}$	$\mathbf{2^{-11}}$	**0.8396**
RBF	2^{5}	2^{-9}	0.8208
RBF	2^{5}	2^{-7}	0.7925
RBF	2^{5}	2^{-5}	0.5849
RBF	2^{5}	2^{-3}	0.5377
RBF	2^{5}	2^{-1}	0.5377
RBF	2^{5}	2^{1}	0.5377
RBF	2^{5}	2^{3}	0.5377
Linear	2^{-5}	-	0.5377
Linear	2^{-3}	-	0.5377
Linear	2^{-1}	-	0.5377
Linear	2^{1}	-	0.6509
Linear	2^{3}	-	0.7547
Linear	2^{5}	-	0.7925

Random Forest (RF)

Split criteria (criterion)	# of trees (estimators)	The minimum # of samples in a leaf node (min_samples_leaf)	Accruacy
Information gain	100	1	0.7453
Information gain	100	2	0.7075
Information gain	100	3	0.7170
Information gain	100	4	0.7170
Information gain	100	5	0.7075
Information gain	300	1	0.7358
Information gain	300	2	0.7170
Information gain	300	3	0.7075
Information gain	300	4	0.7453
Information gain	300	5	0.6981
Information gain	500	1	0.7170
Information gain	500	2	0.7264
Information gain	500	3	0.7358
Information gain	500	4	0.7264
Information gain	500	5	0.7264
Information gain	700	1	0.7075
Information gain	700	2	0.7264
Information gain	700	3	0.7075
Information gain	700	4	0.7264
Information gain	700	5	0.7075
Information gain	900	1	0.7264
Information gain	900	2	0.7170
Information gain	900	3	0.7264
Information gain	900	4	0.7170
Information gain	900	5	0.7170
**Gini impurity**	**100**	**1**	**0.7547**
Gini impurity	100	2	0.7358
Gini impurity	100	3	0.7453
Gini impurity	100	4	0.7358
Gini impurity	100	5	0.7358
Gini impurity	300	1	0.7358
Gini impurity	300	2	0.7264
Gini impurity	300	3	0.7170
Gini impurity	300	4	0.7264
Gini impurity	300	5	0.7170

Gini impurity	500	1	0.7170
Gini impurity	500	2	0.7170
Gini impurity	500	3	0.7453
Gini impurity	500	4	0.7358
Gini impurity	500	5	0.7264
Gini impurity	700	1	0.7264
Gini impurity	700	2	0.7264
Gini impurity	700	3	0.7075
Gini impurity	700	4	0.7358
Gini impurity	700	5	0.7170
Gini impurity	900	1	0.7358
Gini impurity	900	2	0.7358
Gini impurity	900	3	0.7170
Gini impurity	900	4	0.7170
Gini impurity	900	5	0.7264

K-nearest neighbors (KNN)

Weight function (weights)	# of neighbors (n_neighbors)	Accuracy
uniform	5	0.6887
uniform	10	0.6981
uniform	15	0.6698
uniform	20	0.6604
uniform	25	0.6509
distance	5	0.6792
**distance**	**10**	**0.6981**
distance	15	0.6698
distance	20	0.6698
distance	25	0.6509

Decision Tree (DT)

Split criteria (criterion)	The minimum # of samples in a leaf node (min_samples_leaf)	Accruacy
Information gain	1	0.6887
Information gain	2	0.6981
**Information gain**	**3**	**0.7075**
Information gain	4	0.6792
Information gain	5	0.7075
Gini impurity	1	0.6132
Gini impurity	2	0.6226
Gini impurity	3	0.6509
Gini impurity	4	0.6887
Gini impurity	5	0.6415

Supplementary Material S4. Performance comparison of meth-SemiCancer with the baseline methods based on 10-fold cross validation (CV) for COAD, GBM, and THCA cancer. The Boxplot for each cancer shows the distribution of MCCs for the classifiers in 10-fold CV of each cancer subtype prediction.

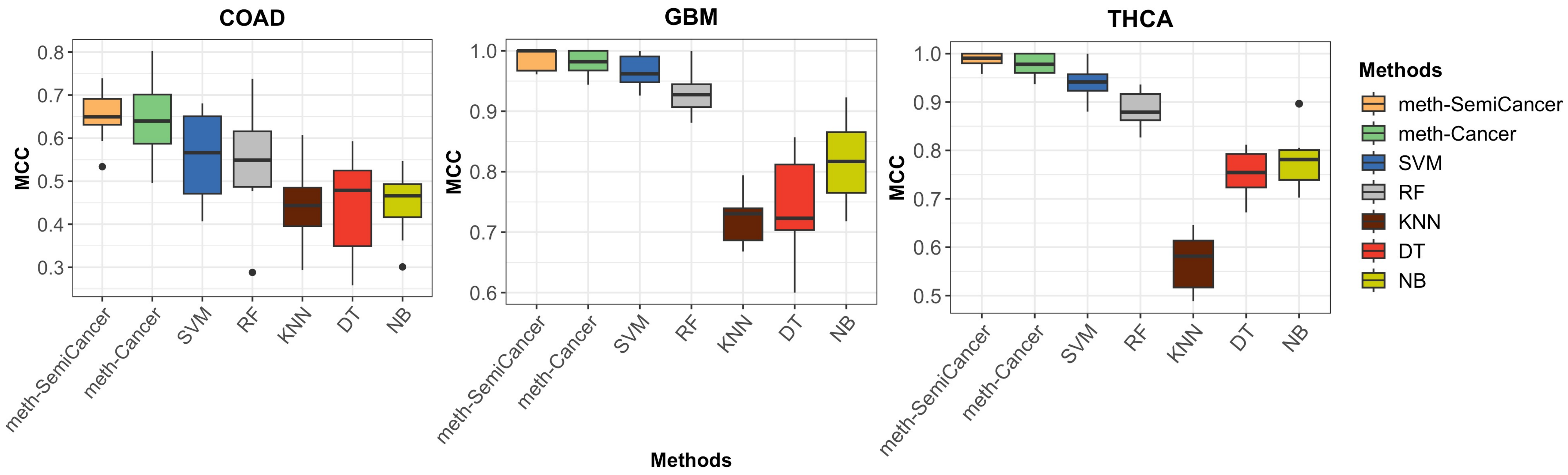

Supplementary Material S5.
The average accuracy, weighted F1-score, and Matthews correlation coefficient (MCC), precision, recall, and Cohen's Kappa results of meth-SemiCancer and the baseline methods from the performance evaluation based on 10-fold cross validation.

[BRCA]

	meth-SemiCancer	meth-Cancer	SVM	RF	KNN	DT	NB
Accuracy	0.836	0.805	0.814	0.715	0.702	0.702	0.617
F1-score	0.824	0.814	0.804	0.672	0.619	0.667	0.645
MCC	0.755	0.719	0.712	0.538	0.536	0.533	0.461
Precision	0.822	0.739	0.756	0.605	0.663	0.522	0.567
Recall	0.725	0.799	0.700	0.492	0.478	0.506	0.592
Kappa	0.743	0.724	0.714	0.551	0.464	0.484	0.450

[COAD]

	meth-SemiCancer	meth-Cancer	SVM	RF	KNN	DT	NB
Accuracy	0.766	0.754	0.678	0.668	0.585	0.588	0.592
F1-score	0.769	0.752	0.678	0.665	0.566	0.592	0.580
MCC	0.689	0.665	0.561	0.548	0.440	0.440	0.446
Precision	0.781	0.741	0.686	0.669	0.622	0.585	0.596
Recall	0.689	0.665	0.673	0.656	0.582	0.586	0.591
Kappa	0.678	0.660	0.555	0.540	0.421	0.436	0.435

[GBM]

	meth-SemiCancer	meth-Cancer	SVM	RF	KNN	DT	NB
Accuracy	0.990	0.985	0.972	0.945	0.767	0.791	0.852
F1-score	0.990	0.985	0.972	0.945	0.758	0.790	0.853
MCC	0.987	0.981	0.964	0.931	0.722	0.736	0.816
Precision	0.991	0.988	0.972	0.948	0.816	0.801	0.859
Recall	0.991	0.984	0.975	0.947	0.796	0.805	0.867
Kappa	0.986	0.981	0.964	0.930	0.704	0.733	0.812

[PRAD]

	meth-SemiCancer	meth-Cancer	SVM	RF	KNN	DT	NB
Accuracy	0.916	0.886	0.856	0.711	0.702	0.753	0.726
F1-score	0.913	0.882	0.841	0.675	0.661	0.739	0.694
MCC	0.888	0.840	0.783	0.561	0.550	0.639	0.590
Precision	0.805	0.811	0.708	0.494	0.529	0.516	0.527
Recall	0.853	0.837	0.701	0.497	0.492	0.531	0.517
Kappa	0.876	0.845	0.777	0.622	0.547	0.594	0.574

[RCC]

	meth-SemiCancer	meth-Cancer	SVM	RF	KNN	DT	NB
Accuracy	0.963	0.946	0.956	0.923	0.883	0.901	0.853
F1-score	0.964	0.948	0.957	0.922	0.859	0.900	0.855
MCC	0.939	0.903	0.917	0.854	0.776	0.812	0.727
Precision	0.942	0.885	0.932	0.966	0.703	0.890	0.838
Recall	0.903	0.964	0.949	0.929	0.661	0.883	0.843
Kappa	0.946	0.909	0.916	0.915	0.765	0.831	0.724

[THCA]

	meth-SemiCancer	meth-Cancer	SVM	RF	KNN	DT	NB
Accuracy	0.990	0.984	0.958	0.918	0.578	0.828	0.841
F1-score	0.990	0.984	0.958	0.918	0.516	0.827	0.839
MCC	0.986	0.977	0.940	0.882	0.569	0.752	0.777
Precision	0.992	0.980	0.953	0.908	0.686	0.805	0.796
Recall	0.988	0.988	0.959	0.900	0.741	0.801	0.826
Kappa	0.986	0.977	0.939	0.880	0.476	0.749	0.772

Supplementary Material S6.

The average accuracy results of meth-SemiCancer under different sample sizes for pseudo-labeling during fine-tuning based on 10-fold cross-validation.

	**Simulation dataset**				
Sample size	0	400	600	800	1000
**BRCA**	0.805	0.830	0.834	0.833	0.837
**PRAD**	0.886	0.897	0.895	0.898	0.910
**RCC**	0.946	0.974	0.979	0.978	0.976

	**Real-studies dataset (GEO)**				
Sample size	0%	40%	60%	80%	100%
**BRCA**	0.805	0.834	0.833	0.835	0.842
**PRAD**	0.886	0.918	0.916	0.916	0.925
**RCC**	0.946	0.969	0.970	0.974	0.968

Supplementary Material S7.

The number of unlabeled samples utilized during training the meth-SemiCancer for each fine-tuning epoch based on the different confidence threshold.

[Confidence Threshold 0.5]

Fine-tuning epoch	The number of unlabeled samples
10	523
20	886
30	649
40	1019
50	850
60	705
70	928
80	851
90	833
100	964
110	825
120	1025
130	1024
140	933
150	1085
160	949
170	1026
180	1082
190	1098
200	1145
210	1134
220	1151
230	1181
240	1183
250	1129
260	1209
270	1196
280	918
290	1098
300	1051
310	1153
320	1149
330	1185
340	1165
350	1184
360	1199
370	1206
380	1215
390	1220
400	1223
410	1223
420	1219
430	1224
440	1224
450	1225
460	1225
470	1225
480	1225
490	1225

500	1225
510	1225
520	1218
530	1175
540	1222
550	1223
560	1225
570	1225
580	1225
590	1225
600	1225
610	1225
620	1225
630	1225
640	1225
650	1208
660	1224
670	1225
680	1224
690	1225
700	1225
710	1225
720	1225
730	1225
740	1225
750	1225
760	1225
770	1225
780	1225
790	1225
800	1225
810	1225
820	1225
830	1225
840	1225
850	1225
860	1225
870	1225
880	1225
890	1225
900	1225
910	1225
920	1225
930	1225
940	1225
950	1225
960	1225
970	1225
980	1225
990	1225
1000	1225
1010	1224
1020	1225
1030	1225
1040	1225
1050	1225

1060	1225
1070	1225
1080	1176
1090	1109
1100	1202
1110	1203
1120	1196
1130	1197
1140	1210
1150	1218
1160	1219
1170	1220
1180	1221
1190	1222
1200	1221
1210	1222
1220	1221
1230	1224
1240	1223
1250	1222
1260	1223
1270	1224
1280	1224
1290	1225
1300	1225
1310	1224
1320	1224
1330	1225
1340	1225
1350	1225
1360	1225
1370	1225
1380	1225
1390	1225
1400	1225
1410	1225
1420	1225
1430	1225
1440	1225
1450	1225
1460	1225
1470	1225
1480	1225
1490	1225
1500	1225
1510	1225
1520	1225
1530	1225
1540	1225
1550	1225
1560	1225
1570	1225
1580	1225
1590	1225
1600	1225
1610	1225

1620	1225
1630	1225
1640	1225
1650	1225
1660	1225
1670	1225
1680	1225
1690	1225
1700	1225
1710	1225
1720	1225
1730	1224
1740	1225
1750	1225
1760	1225
1770	1225
1780	1225
1790	1225
1800	1225
1810	1225
1820	1225
1830	1225
1840	1225
1850	1225
1860	1225
1870	1225
1880	1225
1890	1225
1900	1225
1910	1225
1920	1225
1930	1225
1940	1225
1950	1225
1960	1225
1970	1225
1980	1225
1990	1225
2000	1225
2010	1225
2020	1225
2030	1225
2040	1225
2050	1225
2060	1225
2070	1157
2080	1168
2090	1188
2100	1191
2110	1210
2120	1214
2130	1221
2140	1222
2150	1225
2160	1224
2170	1225

2180	1225
2190	1225
2200	1225
2210	1218
2220	1223
2230	1225
2240	1224
2250	1224
2260	1225
2270	1225
2280	1225
2290	1225
2300	1225
2310	1225
2320	1217
2330	1225
2340	1225
2350	1225
2360	1225
2370	1225
2380	1225
2390	1225
2400	1225
2410	1225
2420	1225
2430	1225
2440	1225
2450	1225
2460	1225
2470	1225
2480	1225
2490	1225
2500	1225
2510	1197
2520	1224
2530	1206
2540	1221
2550	1224
2560	1223
2570	1225
2580	1225
2590	1225
2600	1225
2610	1225
2620	1225
2630	1225
2640	1225
2650	1225
2660	1225
2670	1225
2680	1225
2690	1225
2700	1225
2710	1225
2720	1225
2730	1225

2740	1225
2750	1225
2760	1225
2770	1225
2780	1225
2790	1225
2800	1225
2810	1225
2820	1225
2830	1225
2840	1225
2850	1225
2860	1225
2870	1225
2880	1225
2890	1225
2900	1225
2910	1225
2920	1225
2930	1225
2940	1225
2950	1225
2960	1225
2970	1225
2980	1225
2990	1225
3000	1225

[Confidence Threshold 0.6]

Fine-tuning epoch	The number of unlabeled samples
10	772
20	467
30	304
40	721
50	550
60	466
70	554
80	571
90	533
100	829
110	744
120	717
130	908
140	934
150	809
160	859
170	869
180	1014
190	1036
200	909
210	1092
220	1090
230	1097
240	1068
250	1071

260	905
270	1136
280	1131
290	1156
300	1065
310	1165
320	1178
330	1179
340	1113
350	1181
360	1193
370	1174
380	1134
390	1147
400	1183
410	1218
420	1193
430	1203
440	1220
450	1219
460	1221
470	1222
480	1216
490	1222
500	1221
510	1222
520	1220
530	1225
540	1225
550	1224
560	1224
570	1177
580	1225
590	1224
600	1224
610	1225
620	1224
630	1224
640	1225
650	1225
660	1225
670	1225
680	1225
690	1225
700	1225
710	1225
720	1225
730	1223
740	1225
750	1225
760	1225
770	1225
780	1225
790	1225
800	1225
810	1225

820	1225
830	1225
840	1225
850	1225
860	1221
870	1225
880	1225
890	1225
900	1217
910	1225
920	1225
930	1225
940	1225
950	1224
960	1135
970	1225
980	1225
990	1225
1000	1225
1010	1225
1020	1225
1030	1225
1040	1225
1050	1224
1060	1225
1070	1225
1080	1225
1090	1225
1100	1224
1110	1225
1120	1225
1130	1225
1140	1225
1150	1225
1160	1225
1170	1225
1180	1225
1190	1225
1200	1224
1210	1223
1220	1225
1230	1225
1240	1221
1250	1225
1260	1225
1270	1225
1280	1225
1290	1225
1300	1116
1310	1123
1320	1134
1330	1171
1340	1203
1350	1209
1360	1218
1370	1217

1380	1221
1390	1222
1400	1223
1410	1224
1420	1224
1430	1224
1440	1225
1450	1225
1460	1225
1470	1225
1480	1225
1490	1225
1500	1225
1510	1225
1520	1225
1530	1225
1540	1225
1550	1225
1560	1225
1570	1225
1580	1225
1590	1225
1600	1225
1610	1225
1620	1225
1630	1225
1640	1225
1650	1225
1660	1225
1670	1225
1680	1225
1690	1225
1700	1225
1710	1225
1720	1225
1730	1225
1740	1225
1750	1225
1760	1225
1770	1225
1780	1225
1790	1225
1800	1225
1810	1225
1820	1225
1830	1225
1840	1225
1850	1225
1860	1225
1870	1225
1880	1225
1890	1225
1900	1225
1910	1225
1920	1225
1930	1225

1940	1225
1950	1225
1960	1225
1970	1225
1980	1225
1990	1225
2000	1225
2010	1225
2020	1225
2030	1184
2040	1223
2050	1223
2060	1225
2070	1225
2080	1225
2090	1225
2100	1225
2110	1225
2120	1225
2130	1225
2140	1225
2150	1225
2160	1225
2170	1225
2180	1225
2190	1225
2200	1225
2210	1225
2220	1225
2230	1225
2240	1225
2250	1225
2260	1225
2270	1225
2280	1225
2290	1225
2300	1225
2310	1225
2320	1225
2330	1225
2340	1225
2350	1225
2360	1225
2370	1225
2380	1225
2390	1225
2400	1225
2410	1225
2420	1225
2430	1225
2440	1225
2450	1225
2460	1225
2470	1225
2480	1225
2490	1225

2500	1225
2510	1225
2520	1225
2530	1225
2540	1225
2550	1225
2560	1225
2570	1225
2580	1225
2590	1225
2600	1225
2610	1225
2620	1225
2630	1225
2640	1225
2650	1225
2660	1225
2670	1225
2680	1225
2690	1225
2700	1225
2710	1225
2720	1225
2730	1225
2740	1225
2750	1225
2760	1225
2770	1192
2780	1170
2790	1125
2800	1163
2810	1154
2820	1189
2830	1203
2840	1210
2850	1200
2860	1216
2870	1219
2880	1222
2890	1225
2900	1225
2910	1225
2920	1225
2930	1225
2940	1225
2950	1225
2960	1225
2970	1225
2980	1225
2990	1225
3000	1225

[Confidence Threshold 0.7]

Fine-tuning epoch	The number of unlabeled samples
10	666

20	506
30	148
40	265
50	499
60	321
70	291
80	462
90	345
100	321
110	509
120	479
130	541
140	592
150	608
160	644
170	723
180	716
190	882
200	904
210	808
220	924
230	1013
240	867
250	947
260	905
270	992
280	873
290	1008
300	1052
310	1017
320	997
330	1058
340	1072
350	1117
360	1144
370	1099
380	1148
390	1141
400	999
410	1091
420	1129
430	1176
440	1167
450	1190
460	1137
470	1196
480	1196
490	1202
500	1191
510	1207
520	1211
530	1207
540	1175
550	1209
560	1170
570	1207

580	1107
590	1212
600	1205
610	1215
620	1214
630	1216
640	1214
650	1219
660	1219
670	1219
680	1219
690	1209
700	1206
710	1213
720	1128
730	1219
740	1219
750	1120
760	1217
770	1222
780	1221
790	1199
800	1221
810	1217
820	1076
830	1223
840	1223
850	1222
860	1221
870	1221
880	1222
890	1217
900	1223
910	1224
920	1224
930	1224
940	1224
950	1222
960	1224
970	1220
980	1224
990	1224
1000	1219
1010	1224
1020	1224
1030	1224
1040	1224
1050	1224
1060	1211
1070	1225
1080	1225
1090	1225
1100	1225
1110	1224
1120	1225
1130	1224

1140	979
1150	1144
1160	1172
1170	1186
1180	1199
1190	1208
1200	1209
1210	1202
1220	1218
1230	1219
1240	1218
1250	1221
1260	1220
1270	1223
1280	1224
1290	1224
1300	1224
1310	1224
1320	1224
1330	1224
1340	1224
1350	1224
1360	1224
1370	1224
1380	1224
1390	1224
1400	1224
1410	1224
1420	1224
1430	1224
1440	1225
1450	1225
1460	1225
1470	1225
1480	1225
1490	1225
1500	1225
1510	1225
1520	1225
1530	1225
1540	1225
1550	1225
1560	1220
1570	1225
1580	1225
1590	1225
1600	1225
1610	1225
1620	1225
1630	1225
1640	1225
1650	1225
1660	1225
1670	1225
1680	1225
1690	1225

1700	1225
1710	1225
1720	1225
1730	1225
1740	1225
1750	1225
1760	1224
1770	1225
1780	1225
1790	1225
1800	1225
1810	1225
1820	1225
1830	1225
1840	1225
1850	1225
1860	1225
1870	1225
1880	1225
1890	1225
1900	968
1910	1161
1920	1110
1930	1146
1940	1191
1950	1195
1960	1185
1970	1210
1980	1209
1990	1221
2000	1223
2010	1223
2020	1225
2030	1225
2040	1225
2050	1225
2060	1225
2070	1225
2080	1225
2090	1225
2100	1225
2110	1225
2120	1225
2130	1225
2140	1225
2150	1225
2160	1225
2170	1225
2180	1225
2190	1225
2200	1225
2210	1225
2220	1225
2230	1225
2240	1225
2250	1225

2260	1225
2270	1225
2280	1225
2290	1225
2300	1225
2310	1225
2320	1225
2330	1225
2340	1225
2350	1225
2360	1225
2370	1225
2380	1225
2390	1225
2400	1225
2410	1225
2420	1225
2430	1225
2440	1201
2450	1225
2460	1225
2470	1225
2480	1225
2490	1225
2500	1225
2510	1225
2520	1225
2530	1225
2540	1225
2550	1225
2560	1225
2570	1225
2580	1225
2590	1225
2600	1225
2610	1225
2620	1225
2630	1225
2640	1225
2650	1225
2660	1225
2670	1225
2680	1225
2690	1225
2700	1225
2710	1225
2720	1225
2730	1225
2740	1225
2750	1225
2760	1225
2770	1225
2780	1225
2790	1225
2800	1225
2810	1225

2820	1225
2830	1225
2840	1225
2850	1225
2860	1225
2870	1225
2880	1225
2890	1225
2900	1225
2910	1225
2920	1225
2930	1225
2940	1225
2950	1225
2960	1225
2970	1225
2980	1225
2990	1225
3000	1225

[Confidence Threshold 0.8]

Fine-tuning epoch	The number of unlabeled samples
10	492
20	344
30	195
40	106
50	191
60	260
70	149
80	120
90	310
100	426
110	288
120	232
130	361
140	384
150	336
160	425
170	541
180	581
190	574
200	517
210	727
220	675
230	632
240	749
250	838
260	676
270	875
280	800
290	711
300	877
310	901
320	944
330	944

340	964
350	947
360	991
370	998
380	985
390	1005
400	932
410	923
420	964
430	1058
440	765
450	1055
460	1087
470	1042
480	1100
490	1107
500	1081
510	1115
520	1119
530	1119
540	1122
550	1121
560	1125
570	905
580	1113
590	1080
600	1085
610	1137
620	1146
630	1137
640	1078
650	1154
660	1135
670	1160
680	1159
690	1136
700	1178
710	1178
720	1171
730	1156
740	1186
750	1185
760	1154
770	1179
780	532
790	928
800	656
810	896
820	748
830	1076
840	1128
850	1122
860	1124
870	1138
880	1190
890	1162

900	1193
910	1196
920	1195
930	1138
940	1198
950	1196
960	1200
970	1200
980	1204
990	1194
1000	1197
1010	1203
1020	1186
1030	1202
1040	1203
1050	1204
1060	1203
1070	1203
1080	1204
1090	1205
1100	1203
1110	1205
1120	1205
1130	1205
1140	1205
1150	1206
1160	1204
1170	1208
1180	1205
1190	1205
1200	1206
1210	1206
1220	1206
1230	1208
1240	1208
1250	1209
1260	1208
1270	1209
1280	1209
1290	1210
1300	1210
1310	1209
1320	1210
1330	1210
1340	1210
1350	1210
1360	1210
1370	1210
1380	1210
1390	1208
1400	1210
1410	1210
1420	1210
1430	1210
1440	1210
1450	1210

1460	1209
1470	1211
1480	1211
1490	1211
1500	1211
1510	1211
1520	1211
1530	1211
1540	1211
1550	1211
1560	1211
1570	1211
1580	1211
1590	1211
1600	1211
1610	1211
1620	1211
1630	1211
1640	1211
1650	1212
1660	1215
1670	1216
1680	1216
1690	1216
1700	1216
1710	1216
1720	1216
1730	1215
1740	1216
1750	1216
1760	1216
1770	1216
1780	1216
1790	1216
1800	1216
1810	1216
1820	1216
1830	1216
1840	1216
1850	1216
1860	1216
1870	1218
1880	1218
1890	1218
1900	1218
1910	1219
1920	1219
1930	1219
1940	1219
1950	1219
1960	1219
1970	1220
1980	1221
1990	1221
2000	697
2010	993

2020	1071
2030	1154
2040	1168
2050	1178
2060	1179
2070	1187
2080	1182
2090	1184
2100	1200
2110	1201
2120	1210
2130	1212
2140	1215
2150	1217
2160	1214
2170	1216
2180	1217
2190	1209
2200	1217
2210	1217
2220	1217
2230	1218
2240	1218
2250	1218
2260	1218
2270	1218
2280	1218
2290	1218
2300	1218
2310	1218
2320	1218
2330	1218
2340	1218
2350	1218
2360	1166
2370	1213
2380	1215
2390	1185
2400	1219
2410	1219
2420	1222
2430	1222
2440	1222
2450	1222
2460	1222
2470	1222
2480	1222
2490	1222
2500	1222
2510	1222
2520	1222
2530	1222
2540	1222
2550	1222
2560	1222
2570	1222

2580	1222
2590	1222
2600	1222
2610	1223
2620	1223
2630	1223
2640	1223
2650	1223
2660	1223
2670	1223
2680	1223
2690	1223
2700	1222
2710	1223
2720	1223
2730	1223
2740	1223
2750	1223
2760	1223
2770	1223
2780	1223
2790	1223
2800	1223
2810	1224
2820	1224
2830	1224
2840	1224
2850	1224
2860	1224
2870	1224
2880	1224
2890	1224
2900	1224
2910	1224
2920	1224
2930	1224
2940	1224
2950	1224
2960	1224
2970	1224
2980	1224
2990	1224
3000	1224

[Confidence Threshold 0.9]

Fine-tuning epoch	The number of unlabeled samples
10	80
20	79
30	29
40	106
50	57
60	186
70	45
80	326
90	106

100	342
110	122
120	324
130	102
140	353
150	117
160	305
170	133
180	576
190	150
200	621
210	279
220	358
230	376
240	362
250	735
260	561
270	336
280	530
290	519
300	522
310	526
320	890
330	905
340	840
350	821
360	939
370	619
380	948
390	941
400	943
410	947
420	890
430	932
440	704
450	934
460	965
470	935
480	986
490	625
500	971
510	946
520	905
530	997
540	845
550	988
560	901
570	1010
580	1026
590	1029
600	760
610	1002
620	976
630	1034
640	1032
650	1003

660	967
670	1047
680	1050
690	1053
700	1053
710	1055
720	1050
730	1062
740	1049
750	1055
760	1039
770	1056
780	1063
790	1035
800	1058
810	1054
820	1065
830	1069
840	949
850	1072
860	1072
870	1077
880	1069
890	1078
900	1067
910	1079
920	1077
930	1081
940	1079
950	1067
960	1088
970	1090
980	1088
990	964
1000	763
1010	540
1020	506
1030	851
1040	715
1050	1044
1060	1079
1070	1082
1080	1091
1090	1092
1100	1129
1110	1121
1120	1119
1130	1117
1140	1137
1150	1142
1160	1140
1170	1145
1180	1142
1190	1146
1200	1146
1210	1149

1220	1149
1230	1149
1240	1152
1250	1148
1260	1156
1270	1158
1280	1157
1290	1157
1300	1158
1310	1158
1320	1159
1330	1162
1340	1168
1350	1163
1360	1167
1370	1165
1380	1169
1390	1168
1400	1169
1410	1170
1420	1163
1430	1170
1440	1170
1450	1170
1460	1170
1470	1170
1480	1170
1490	1170
1500	1171
1510	1171
1520	1171
1530	1171
1540	1172
1550	1172
1560	1173
1570	1172
1580	1172
1590	1137
1600	1173
1610	1173
1620	1174
1630	1174
1640	1174
1650	1174
1660	1083
1670	1159
1680	1162
1690	1175
1700	1178
1710	1177
1720	1178
1730	1178
1740	1181
1750	1183
1760	1183
1770	1183

1780	1183
1790	1183
1800	1183
1810	1183
1820	1184
1830	1184
1840	1166
1850	1181
1860	1188
1870	1191
1880	1192
1890	1189
1900	1192
1910	1199
1920	1197
1930	1197
1940	842
1950	421
1960	710
1970	719
1980	921
1990	956
2000	996
2010	1012
2020	1025
2030	1032
2040	1041
2050	1049
2060	1057
2070	1052
2080	1075
2090	1064
2100	1057
2110	1065
2120	1070
2130	1081
2140	1082
2150	1081
2160	1093
2170	1095
2180	1096
2190	1097
2200	1097
2210	1097
2220	1104
2230	1103
2240	1105
2250	1106
2260	1108
2270	1109
2280	1123
2290	1114
2300	1112
2310	1126
2320	1126
2330	1123

2340	1120
2350	1132
2360	1125
2370	1148
2380	1136
2390	1128
2400	1136
2410	1141
2420	1134
2430	1145
2440	1147
2450	1148
2460	1144
2470	1159
2480	1159
2490	1142
2500	1151
2510	1177
2520	1186
2530	1178
2540	1187
2550	1186
2560	1184
2570	1185
2580	1181
2590	1192
2600	1188
2610	1188
2620	1187
2630	1188
2640	1203
2650	1192
2660	1203
2670	1194
2680	1197
2690	1196
2700	1203
2710	1206
2720	1206
2730	1208
2740	1191
2750	1192
2760	1156
2770	1093
2780	1207
2790	1212
2800	1193
2810	1216
2820	1216
2830	1214
2840	1153
2850	1209
2860	1216
2870	1215
2880	1217
2890	1217

2900	1207
2910	1219
2920	1219
2930	1218
2940	1219
2950	1219
2960	1219
2970	1218
2980	1219
2990	1219
3000	1219
